# Development of Kaempferol-Loaded Gelatin Nanoparticles for the Treatment of Corneal Neovascularization in Mice

**DOI:** 10.3390/pharmaceutics11120635

**Published:** 2019-11-28

**Authors:** Yu-Lun Chuang, Hsu-Wei Fang, Aditya Ajitsaria, Ko-Hua Chen, Chen-Ying Su, Guei-Sheung Liu, Ching-Li Tseng

**Affiliations:** 1 Graduate Institute of Biomedical Materials and Tissue Engineering, College of Biomedical Engineering, Taipei Medical University, Taipei 11031, Taiwan; a3750585@gmail.com; 2 Department of Chemical Engineering and Biotechnology, National Taipei University of Technology, Taipei 10608, Taiwan; hsuweifang@gmail.com (H.-W.F.); chenying.su@gmail.com (C.-Y.S.); 3 Institute of Biomedical Engineering and Nanomedicine, National Health Research Institutes, Miaoli County 35053, Taiwan; 4 International Ph.D. Program for Cell Therapy and Regeneration Medicine, School of Medicine, College of Medicine, Taipei Medical University, Taipei 11031, Taiwan; aditya.ajitsaria.7@gmail.com; 5 Department of Ophthalmology, Taipei Veterans General Hospital, Taipei 11217, Taiwan; khchen7637@gmail.com; 6 Department of Ophthalmology, School of Medicine, College of Medicine, Taipei Medical University, Taipei 11031, Taiwan; 7 Menzies Institute for Medical Research, University of Tasmania, Hobart, TAS 7000, Australia; rickliu0817@gmail.com; 8 Ophthalmology, Department of Surgery, University of Melbourne, East Melbourne, VIC 3002, Australia; 9 International Ph.D. Program in Biomedical Engineering, College of Biomedical Engineering, Taipei Medical University, Taipei 11031, Taiwan; 10 Research Center of Biomedical Device, College of Biomedical Engineering, Taipei Medical University, Taipei 11031, Taiwan

**Keywords:** gelatin nanoparticles, kaempferol, corneal neovascularization, anti-angiogenesis, human umbilical vascular endothelial cells (HUVECs)

## Abstract

Cornea is the transparent layer in front of the eye that does not contain blood vessels. Among eye diseases, corneal neovascularization (NV) is one of the major causes of vision loss, since it can also lead to blindness. An herbal extraction containing flavonoid, kaempferol (KA), with antiangiogenic effect was chosen as a candidate drug for inhibited vessel formation. The use of nanomedicine has led to higher drug bioavailability and slow release of the drug as an effective therapeutic formulation in ocular drug delivery. In this study, we prepared gelatin nanoparticles (GNP) with kaempferol encapsulation (GNP-KA) for corneal NV treatment by topical delivery, i.e., eye drops. We found that GNP with/without KA loading was in the size of 85−150 nm, and its zeta potential was around 22−26 mV. The KA entrapment rate of GNP-KA was around 90−98%, and the loading rate was about 4.6%. The TEM results clearly indicated the GNP-KA NPs to be round spheres. The *in vitro* test involved the adoption of human umbilical vein endothelial cells (HUVECs) for coculture with these nanoparticles. From WST-8 assay, and cell migration examinations, it was evident that GNP-KA had the capacity to inhibit the cell viability and function of HUVECs. The results from *in vivo* tests such as ocular vessels observation, hematoxylin & eosin (H&E) stain, and metalloproteinases (MMP)/vascular endothelial growth factor (VEGF) quantification revealed the mice’s eyes with corneal NV treated by eye drops containing GNP-KA once daily for 7 days had better therapeutic effects with less vessels in-growths in the cornea, compared to the KA solution group by reducing the production of MMP and VEGF in the cornea. Therefore, we expected to achieve a comfortable treatment with a simple method using nanomedicine (GNP-KA) as ophthalmological agent delivered as eye drops.

## 1. Introduction

Corneal disease is a significant global health problem, with visual scarring accounting for about 5% of the blindness worldwide which approximates to 4.9 million people making it the second most common cause of blindness after cataract [1,2,3,4]. Corneal neovascularization (NV) is usually the formation of new blood vessels in the cornea which may be associated with physical damage, chemical burns, microbial infections and inflammation, hypoxia, and limbal barrier function. A continual worsening of corneal NV may lead to partial or complete blindness [1,2,3]. Corneal transplantation is reserved for the blind cases; however, vascularization in the transplanted cornea remains one of the major risk factors for transplant failure. Corneal NV generally implies to a neovascularization of the cornea from the edge to the middle, a result of an imbalance between vascular epithelium growth factor (VEGF) and anti-angiogenic factors like angiostatin and endostatin [2,5]. It also involves the upregulation of angiogenic factors such as metalloproteinases (MMP) causing a degradation of the extracellular matrix and the basement membrane leading to the remaining proteolytic enzymes to enter the space between the epithelium and the corneal stroma[6]. Under such conditions, the corneal epithelial cells and macrophages produce VEGF and b-Fibroblast growth factor (bFGF) which then enter the space between the corneal epithelium and the stroma through MMPs and stimulate angiogenesis in the cornea [2,7].

The traditional pharmaceutics for treating corneal NV is the use of topical steroidal eye drops. However, the long-term use of steroids increases the risk of infection, cataract and glaucoma [8]. The development of anti-angiogenic therapies such as antibodies against the VEGF receptor (Bevacizumab (Avastin^®^) or Aflibercept) has gained some interest in recent years due to its successful use in the treatment of choroidal and retinal NV [2,9,10,11]. These agents have been tested in experimental models and in clinical trials with ocular NV including cornea [9,10,12,13,14]. The clinical outcome from topical anti-VEGF agents tend to be variable with risks of complications such as epithelial erosion and corneal thinning [2,9]. Suppressing VEGF signaling may also have adverse effects on the normal functioning of the eye, in relation to physiological angiogenesis such as the requirement of VEGF for the maintenance of subretinal vasculature and cone photoreceptors [11]. While further study is needed to ensure the suitability of anti-VEGF agents for the treatment of corneal NV, additional or alternative therapeutic approaches should be explored to improve current treatment options. Besides, an ideal therapeutic strategy, comprising of less invasive, reduced risk and long-term inhibition of angiogenesis via lesion targeting is the need of the hour.

As mentioned, many conditions are responsible for corneal NV, therefore, the eyes are usually not only in an angiogenic but also an inflammatory condition. Only anti-VEGF treatment in case of corneal NV is not enough. A dual functional drug with anti-angiogenic and anti-inflammatory capabilities is required for the simultaneous treatment of corneal NV. Flavonoids are one such group of organic compounds, capable of acting on the various mechanisms and etiological factors responsible for the development of different sight threatening ocular diseases [15]. Kaempferol [3,5,7-trihydroxy-2-(4-hydroxyphenyl)-4H-1-benzopyran-4-one], is among one of the most common dietary flavonoids, recently shown to possess antioxidant and antitumor properties [16]. H. Luo revealed Kaempferol to be low in cytotoxicity but still possessing the abilities to inhibit angiogenesis and VEGF expression in human ovarian cancer cells [17]. It was also found to exhibit anti-inflammatory effects and suppress vascular smooth cell migration [18]. Another flavonoid, (−)-Epigallocatechin gallate (EGCG), was revealed to inhibit inflammation in murine dry-eye syndrome [19], and suppress corneal neovascularization due to its anti-inflammatory and anti-angiogenic effect when targeted on the ocular surface by topical delivery [20]. The inhibitory effects of Kaempferol in-vitro is through endothelial cells induced apoptosis [18]. Experiments in zebrafish also concluded that kaempferol could reduce the expression of VEGFR-2 to inhibit angiogenesis [21]. Up until now, few studies focus on using Kaempferol for treating abnormal vessel growth in eyes including corneal neovascularization. Therefore, we think that Kaempferol, a bifunctional agent with anti-angiogenesis & anti-inflammation effect can be used for ocular neovascularization treatment by topical delivery.

Nowadays, ocular drug delivery faces numerous obstacles that impede adequate drug delivery and efficacy. Ways to deliver drugs to the ocular region include oral medications by systemic administration, intravitreal injections, intraocular implants and topical eyedrops [22,23]. Topical delivery of drugs or other compounds are preferred due to the ease of administration and patient compliance. Eyedrops are one of the non-invasive and easy to administer ways of topical drug delivery. However, the corneal and conjunctival epithelia serve as a static barrier to topical instillation of drugs, and have low rates of diffusion. Bioavailability is limited by the volume that can be held by the human eye (~30 μL) and the rapid rate of tear turnover (~1 μL/min) [23,24]. Thus, only <5% of an eyedrop–instilled dose is delivered to anterior sections of the eye due to the elimination of the conjunctiva and nasolacrimal duct [24,25]. High concentration dosage is way to solve the problem of low content, but this method is not accurate due to a dramatical variation in the therapeutic concentration in the ocular tissues. These limitations have prompted researchers to develop novel formulations for the treatment of ocular disorders.

**Figure 1:**
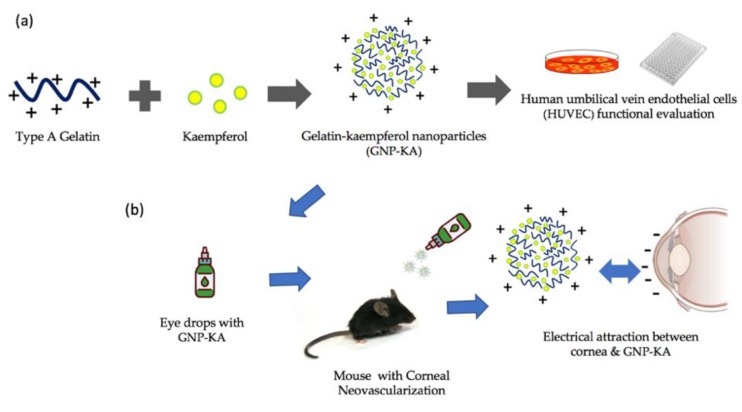
The schematic drawing of this study: (**a**) gelatin nanoparticles with KA loading and its in vitro test in HUVECs, (**b**) the nanomedicine was used as eye drop to treat corneal NV via the electrical attraction between particles and cornea to get good therapeutic effect.

Nanomedicine has also found applications in ophthalmology, by providing safer, less invasive, and cheaper treatment options [26]. Zimmer A et al., stated that a particle size of not more than 10 μm would not act as a foreign body to the eye [27]. The size of nanoparticles should generally lie between 10-1000 nm for a slow release, less irritation as a foreign substance, and lower frequency of administration. The major advantage of using nanomedicine for the treatment of ocular diseases is to achieve targeted drug delivery, controlled release, enhanced bioavailability of topical delivery, and finally improved therapeutic efficacy for ocular disease treatment [24,26]. Nanoparticles made up of various biodegradable polymers like chitosan, gelatin, sodium alginate and hyaluronic acid (HA) can be used effectively for efficient drug delivery to the ocular tissues due to the electrical attraction of nanoparticles with ocular surface [28,29,30,31]. The natamycin (NAT) loaded-nanoparticle consisting of lecithin and chitosan to enhance the NAT precorneal retention, sustain release, high ocular availability at reduce dose and dosing frequency were proofed [29]. Tseng et al., prepared positively and negatively charged gelatin nanoparticles (GNP) with TAMRA-GNP particles and investigated their delivery rates in rabbit cornea showing that the GNP was successful in extending the residence time of the drug in the ocular surface [28]. In this study, a natural polymer derived from collagen, gelatin is used as the raw material to prepare nanoparticles. The main focus of this study is the use of kaempferol loaded in GNP (GNP-KA) to investigate their therapeutic effect on corneal NV in an alkali burning eye model in mice. The schematic drawing of this study is revealed in Figure 1.

## 2. Materials and Methods 

### 2.1. Nanoparticle Preparation

The gelatin nanoparticles (GNP) were prepared by a two-step desolvation method as described previously with some modifications [28,32]. Type A gelatin (bloom 175) was used and purified by acetone for first desolvation followed by dissolution in hot water at final concentrations of 1% (weight/volume, *w*/*v*) and 2% (*w*/*v*). Kaempferol (KA) was subsequently dissolved in DMSO in the storage concentration of 10 mg/mL separately, and then 50 μL of KA solution was added into the gelatin solution (1mL). Acetone was added again dropwise to form. Various parameters for prepared kaempferol (KA) loaded GNP (GNP-KA) were tested including the change of concentration and pH values of gelatin solution; cross-linked by different concentration of glutaraldehyde, and the stirring times for the crosslinking reactions were evaluated. After particle preparation, the remaining organic solvent was evaporated using a rotary evaporator (EYELA, Tokyo, Japan), and the resultant nanoparticles were stored at 4 °C for further examination.

### 2.2. Characterization of GNP-KA

After the preparation of KA loaded GNPs, the dynamic light scattering analyser (DLS) (ZS90 Plus; Malvern, UK) at 25 °C with scattering light at 90 degrees and 180 s was used for measuring the particle size. The surface charge of nanoparticles was also examined by the DLS in the zeta sizer mode. The structure and morphology of nanoparticle was conducted through a transmission electron microscope (TEM, Hitachi HT-7700, Tokyo, Japan) at an acceleration voltage of 75.0 kV. Nanoparticles (GNP or GNP-KA) were dropped onto carbon coated nickel mesh with post-staining by 0.5% uranium acetate (UA) for TEM sample preparation. Moreover, the chemical components for each material were detected by Fourier transform infrared spectroscopy (FT-IR 6200 instrument, Jasco, Tokyo, Japan). Before testing, the lyophilized nanoparticle powder was mixed with potassium bromide (KBr), then used in the FTIR examination with the scanning range between 400–4000 cm^−1^. The KA concentration was quantified using high performance liquid chromatography (HPLC). A C18 column (ZORBAX, Eclipse plus-5 μm, Agilent, Santa Clara, CA, USA) and the mobile phase included methanol, acetonitrile, water and acetic acid in the volume ratio of 40:20:39:1 was used; the flow rate of mobile phase was 1 mL/min. The KA was identified at the 350 nm UV. A series KA concentration from 1–500 μg/mL as prepared and measured as standard curve. Encapsulation efficiency (EE) is calculated as the final drug content compared with the initial added drug content; the loading rate (LR) is calculated as the final drug content in the whole nanomedicine base.

### 2.3. In Vitro Test

#### 2.3.1. Cell Viability Test

Human umbilical vascular cells were used in this study. HUVECs were maintained and cultured in M199 medium with 10% fetal bovine serum (FBS)/1x penicillin-streptomycin-neomycin (PSN) antibiotic mixture addition, supplemented with endothelia cells growth supplement ECGS (30 µg/mL) and heparin (25 U/mL). Before HUVECs were seeded, the culture dishes or plates were need to be precoated with gelatin solution (Type A, B300, 1%) for 30 min. HUVECs (5 × 10^3^ cells per well) were seeded in a 96 well plate; for the next day, the cells were cocultured with variant formulations including GNP, KA solution, and GNP-KA at different GNP/KA concentrations. The cell viability teat was proceeded in three repeat experiments, and there’s 6 wells/per tested group examined for the individual condition. After incubation for 1 and 3 days at 37 °C, the cell viability was examined by using the CCK-8 kit with WST-8 (2-(2-methoxy-4-nitrophenyl)-3- (4-nitrophenyl)-5- (2,4-disulfophenyl)-2H-tetrazolium) reduction assay. WST-8 agent with M199 at 10:1 volume ratio was mixed, and added to the culture solution of a mixed reagent of 110 μL per well, and finally cultured in the incubator for 3 h, then using a microplate reader (Multiskan GO Microplate Spectrophotometer, Thermo Scientific, Waltham, MA, USA) tested at 450 nm. The percentage of viable cells was calculated in comparison to control cells, cultured with medium only.

#### 2.3.2. Cell Migration Test 

Angiogenesis not only combines cell growth but is also accompanied by cell migration for cell growth. HUVECs (2 × 10^5^ cells/well) were seeded into a 24-well plate with 0.1% gelatin pre-coating and incubated with complete medium at 37 °C and 5% CO_2_ overnight, using a 200-µL tip to scrape on the cell layer to form a gap between cells. A line of the cell layer was scrapped by using a 200-µL tip followed by three washes with PBS. Fresh medium containing different formulations including KA solution, GNP, and GNP-KA at different KA concentration was added to the cells. Three selected views along the scraped line in each well were photographed by using an inverted microscope ((DMi8, inverted fluorescence microscope, Leica, Wetzlar, Germany). Cells incubated with fresh medium were used as control. After 2, 6, 8, and 18 h of incubation, images were captured at 40× magnification. The average scraped area in each well was measured and the change in the area for each experimental condition was compared with that of the control. The cell space and distance in different groups were measured and compared using Image J Quantitative Analysis.

### 2.4. In Vivo Test

Male mice (C57BL/6J, 8 to 14 weeks old) were used in this study. The experimental procedure was approved by the Institutional Animal Care and Use Committee (IACUC) of Taipei Medical University (IACUC approval no. LAC-2015-0328, 1 July 2016).

#### 2.4.1. Retention of Nanoparticles on the Ocular Surface

The fluorescent dye, (5-(6)-carboxytetramethylrhodamine succinimidyl ester (TAMRA), Life technologies (Eugene, OR, USA) was also loaded into the GNP and GNP-KA nanoparticles for tracing them in the eyes. C57BL/6 mice were used for live monitoring by an in vivo imaging system (IVIS-200, Alameda, CA, USA). The mice were anesthetized, and the eye drops with fluorescent NPs were directly dosed on the eye (5 μL/eye). The TAMRA (dye solution), GNP and GNP-KA as eyedrops were diluted by saline and adjusted into the same TAMRA concentration for comparison. Mice’s eyes with fluorescence were photographed, and the subtraction of tissue autofluorescence to reduce the background noise was adopted to improve sensitivity. The fluorescent intensity compared with the initial intensity after dosing was compared in different time intervals.

#### 2.4.2. Corneal NV Mice Model Establishment

Seventy-two mice were used in this study for three repeated animal tests, and the mice number for each group (normal, PBS, GNP, KA, and GNP-KA) was six respectively. Briefly, the mice were anesthetized to immobilize them before examination. Next, topical administration of 0.5% Alcaine^®^ (Alcon, Puurs, Belgium) for local anesthesia was performed, followed by pressing the tip of an applicator containing silver nitrate/potassium (25%/75%: Grafco, Atlanta, GA, USA) to the center of the cornea steadily for eight seconds [20,33]. Excess of nitrate was washed by saline solution. Each mouse was treated for only one burnt eye. The KA solution, GNP or GNP-KA colloidals were diluted in PBS as eye drops at a final KA concentration of 7.5 µg/mL, and gelatin concentration of 150 µg/mL. Five microliters of colloidal solution were applied on the mouse eye as eye drops once daily for seven days. The burn stimulus response and the severity of neovascularization were then assessed in anesthetized mice by observation under a hand-held portable slit lamp (SL-17, Kowa Company Ltd., Torrance, CA, USA). The extent of corneal NV was scored according to the blisters with a score range of 0–3 corresponding to as follows: 0—no blisters; 1—slight corneal blisters; 2—moderate increase in the corneal surface blisters; 3—increase in the blister size to the shape of a cone. After a one-week treatment, mice were sacrificed, the eyeballs were carefully removed and placed in cassettes followed by soaking in 10% formalin, then embedded in paraffin for sectioning into 5 µm thickness, and staining by H&E method. The sections were observed by a Slide-based tissue cytometry (Axio Observer Z1, Tissue Gnostics, Vienna, Austria). 

#### 2.4.3. Angiogenetic Factors Examination from Cornea Lysate 

Mice cornea were isolated and weighed. Due to the tiny size of mouse cornea, the cornea from four mice treated in the same group were collected and lysed to get enough amount of protein lysate for examination. Cornea from two batches of animal test were examined. The total proteins from these tissues were extracted with tissue protein extraction buffer (Thermo Fisher Scientific, IL, USA). Bead beater-type homogenizer was operated at 2600 rpm, followed by centrifugation at 10,000 g for 3 min at 4 °C, then the supernatants were collected. Total protein was quantified by Bradford assay (p010, GeneCopoeia, Rockville, MD, USA). Angiogenesis cytokines (MMP-2, MMP-9 and VEGF) content in each group were measured by Quantikine^®^ ELISA kit, Mouse VEGF/total MMP-9/MMP-2 Immunoassay (R&D Systems, Inc. Minneapolis, MN, USA) in triplicate. This experiment was conducted according to manufacturer’s protocol.

### 2.5. Statistical Analysis

Data (mean ± standard deviation (SD) from 2–3 independent experiments were shown, and statistical differences between groups were examined by Student’s t-test or one-way analysis of variance (ANOVA) test using Excel (Microsoft). A probability (*p*) value ≤ 0.05 is statistically significant.

## 3. Results and Discussion

### 3.1. Optimal Parameters for GNP- KA Preparation

Gelatin nanoparticles (GNP) were prepared by a two-step desolvation method as described previously [32]. The final GNP needed to be cross-linked by glutaraldehyde (GA) for stability and rigidity. According to our previous study [28], GA at a final concentration of 0.1% and 0.4% for prepared GNP-KA were tested, the result of which are shown in Table 1. While keeping other operation parameters fixed (at room temperature, pH 2.5 for 2% (*wt.*/*v*) gelatin solution; cross-linking with GA for 16 h overnight); comparing with 0.1% GA, higher GA concentration (0.4%); smaller particles (148 ± 10 nm vs. 281 ± 16 nm) with similar zeta-potential (around +24 mV) can be prepared. But the KA encapsulation efficiency (EE) and loading rate (LR) was higher in the 0.4% GA group (96% ± 2%), therefore, 0.4% GA was chosen for the future tests. Additionally, cross-linking time (1, 3 and 16 h) for the prepared GNP-KA was also evaluated (Table 2). The longer GA reaction time can synthesize smaller sized KA-GNP (90 ± 8 nm) with higher EE (98% ± 1%). Although the size after 3 h (149 ± 10 nm) was bigger than the one after 16 h., the EE for these two time limits had no difference (96% ± 2% vs. +98% ± 1%), so we finally chose 3 h as the time for GA cross-linking reaction to prepare GNP-KA for adequate EE but time saving. Finally, we also liked to increase the KA ratio in nanoparticles; thus, a lower concentration of gelatin solution (1% (*w*/*v*) was tested (Table 3). In the low polymer-based NP, the KA ratio in nanomedicine can be increased to 4.6% with a smaller size (85 ± 8 nm) but still higher EE (95%). According to these results, the 1% (*w*/*v*) gelatin Type A solution at pH 2.5 with KA addition to form nanoparticles, then cross-linked with 0.4% (*v*/*v*) GA for 3 h was the optimal condition for the preparation of GNP-KA as a nanomedicine (85 ± 8 nm) with a positive charge (+25.6 ± 2.1 mV) and a high KA EE (95%± 1%) and LE (4.6% ± 0.1%).

### 3.2. Characterization of GNP-KA by TEM and FT-IR

**Figure 2:**
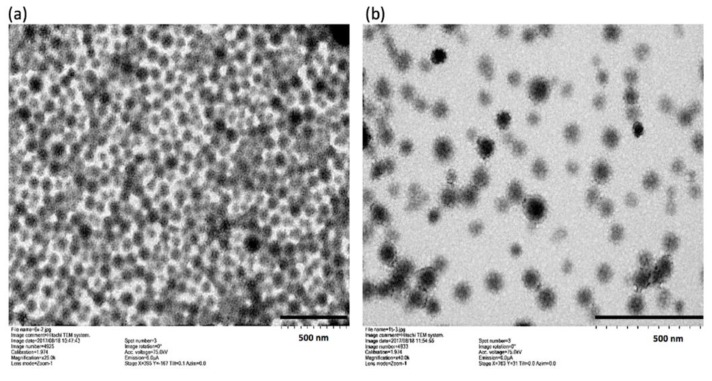

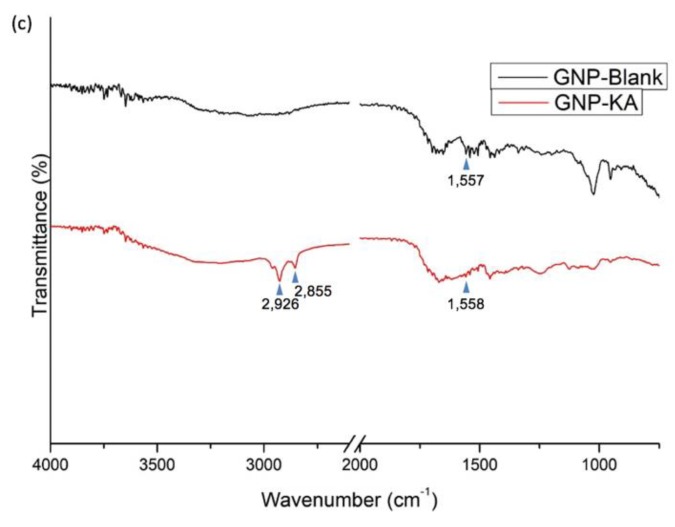
Morphology of GNP-KA examined under TEM. (**a**) 6× dilution (**b**) 15× dilution. and (**c**) FT-IR patterns of GNP, and GNP-KA.

The synthesized GNP-KA presented as round and distinct particles with a spherical structure as assessed by TEM (Figure 2a,b), and its size was around 100 nm in a well dispersed condition showing aggregation, which was comparable to the DLS result. The TEM images of GNP possess similar structures as GNP-KA (Data not shown). The FT-IR spectra of GNP and GNP-KA are shown in Figure 2c. Bands at 1557 cm^−1^ and 1558 cm^−1^ belonging to the amide bond (CN) of gelatin in GNP/GNP-KA were observed. In the pattern of GNP- KA, specific bands at 2855 cm^−1^ and 2926 cm^−1^ of kaempferol (KA) attributed to the phenolic group (OH) [34]. The KA was successfully loaded in GNP.

### 3.3. HUVECs Cell Viability and Migration Capacity Influenced by GNP-KA

**Figure 3:**
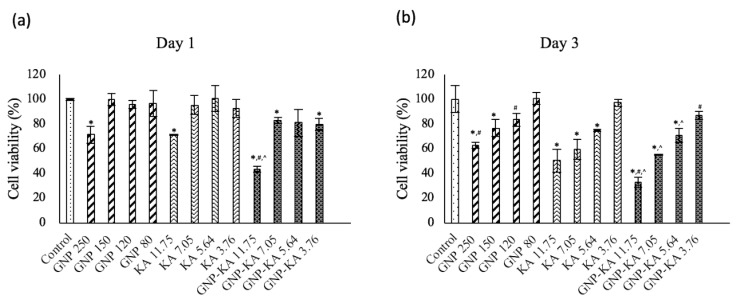
Cell viability of HUVECs treated with various NP formulations at different KA concentrations on (**a**) day 1, and (**b**) day 3. (concentration shows in: μg/mL) (one-way ANOVA, *n* = 6; * *p* < 0.05 compared with control; # *p* < 0.05 compared with KA at the same KA concentration; ^ *p* < 0.05 compared with GNP at the same gelatin concentration.

To check whether KA in different formulations was capable of influencing the cell viability and migration capacity of HUVECs, cells were examined. The KA content was adjusted in the same concentration in KA solution and GNP-KA groups; meanwhile, same gelatin concentration of GNP was also tested to eliminate the effect resulted from the nanoparticles itself. When cells were cultured at different KA concentrations, cell viability was decreased in all groups at day 1 and 3, especially in the GNP-KA groups (Figure 3a,b). The cell viability of the GNP-KA at KA concentration of 11.75 μg/mL (gelatin at 250 μg/mL) was the lowest one 32.81% ± 4.33% (# *p* < 0.05 compared with KA, ^ *p* < 0.05 compared with GNP) in all groups on day 3. However, in the same KA concentration of the KA solution treated one, cell viability was 50.6% ± 9.26%, no so effective as the GNP-KA for inhibiting HUVEC’s viability. In the GNP treated one (same gelatin concentration, 250 μg/mL), the cell viability (62.7% ± 2.13%) was much higher than other groups (# *p* < 0.05 compared with KA, ^ *p* < 0.05 compared with GNP-KA). Despite being biocompatible and biodegradable, gelatin can still cause some toxicity when up taken by cells at a higher concentration (250 μg/mL) in a short period of time. This occurs due to an excessive and undegraded gelatin parts in cells. When using GNP-KA at the lowest KA concentration (3.76 µg/mL) to treat cells, it still can effectively reduce the cell viability (86.9% ± 4.8% (day 3), # *p* < 0.05 compared with KA), but the GNP (80 μg/mL) and KA (3.76 μg/mL) has no influence on cell viability at day 1 and 3 (~100%). The GNP-KA at KA 7.05 μg/mL was adopted for the future test, because at this concentration, GNP-KA can still lower cell viability to 78%, but not the toxicity as the higher treated KA (GNP-KA 11.75 μg/mL, 43%) at day 1.

**Figure 4:**
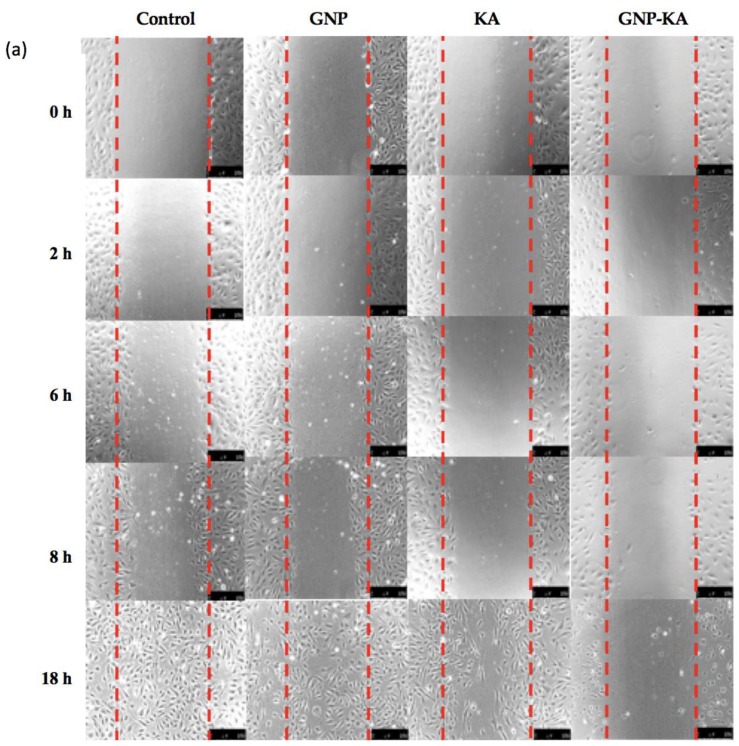

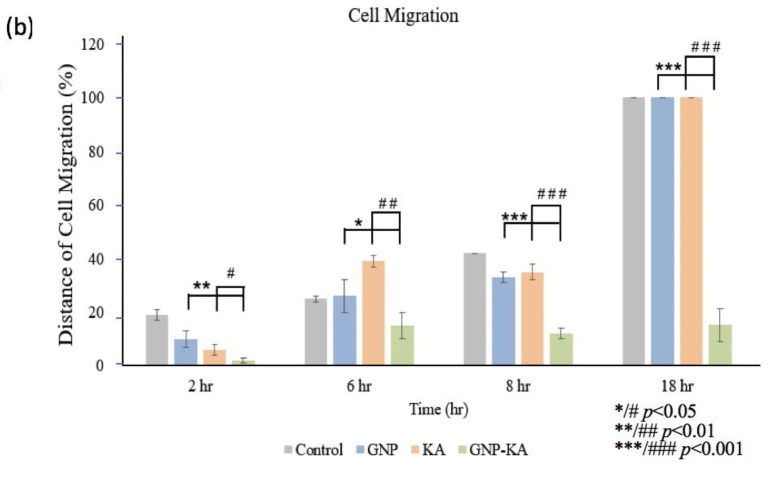
(**a**) Photomicrographs of the cell migration test treated by variant KA formulas, scale bar: 250 µm. (**b**) Quantification results from the images acquired in (**a**). All groups were treated with the same KA and GNP concentration (KA: 7.4 μg/mL, GNP: 150 μg/mL).

The same width of gaps was created by a 200 μL tip on the HUVEC layer in all groups (0 h), and representative photos were chosen for show in Figure 4a treated by variant KA formula (KA 7.4 μg/mL). For the quantification data, 3 photos from each group were used and the cells ratio in the gap area were calculated (Figure 4b). Some HUVECs migrated into the gap after 6 h. After 8 h, many cells already migrated into the gap, the cells in the gap area was 32.8 ± 2.3% in GNP treated one, and 35.3 ± 3.3% gap area was covered with cells in KA group; but the GNP-KA treated one showed less cells in the gap area, just 12.1 ± 1.0% gap area was covered by HUVECs (Figure 4b). Eighteen hours later, there seemed no gap in the control, GNP, and KA groups, almost 100% with cells migration into the gap area. However, the GNP-KA treated group revealed only 15.2 ± 0.5% gap area covered by cells due to cell migration (* compared with GNP: # compared with KA) showing the long-term effect for inhibit cell migration in the KA-loaded nanoparticles (GNP-KA)

### 3.4. Nanoparticles Retain on the Ocular Surface 

**Figure 5:**
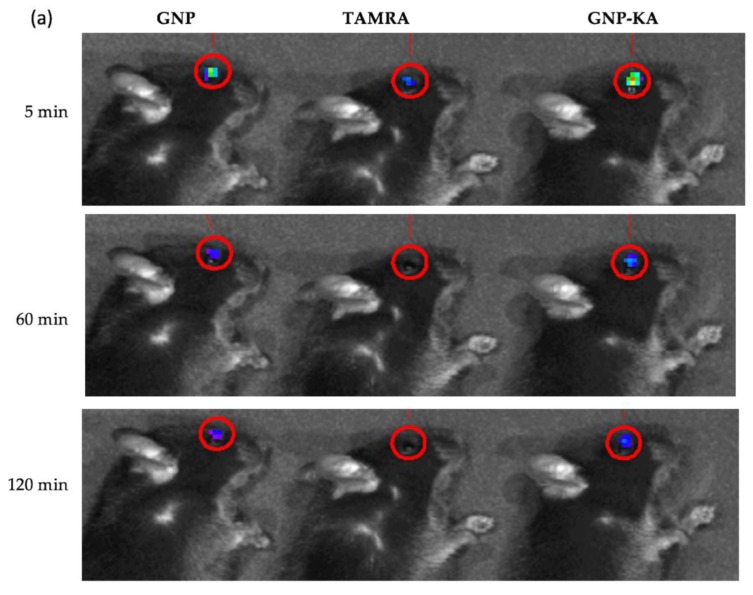

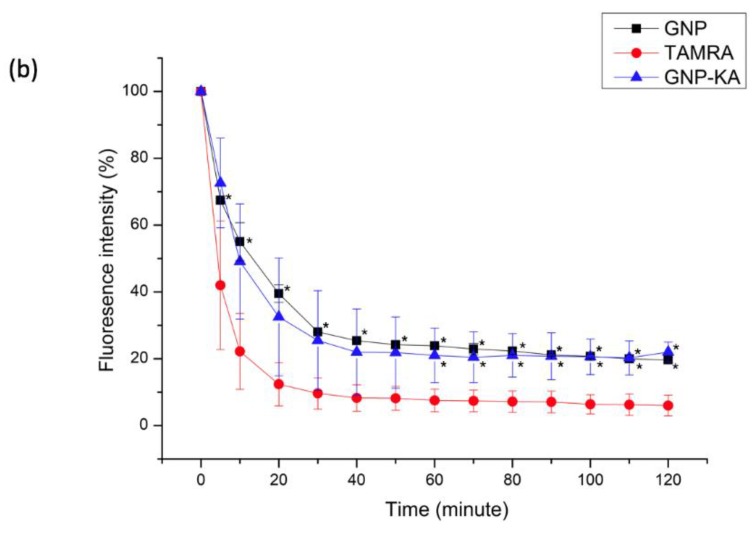
(**a**) Fluorescent dye/particles accumulated on the eye after 5, 60, and 120 min dosing treated different formulations: TAMRA dye solution, GNP, and GNP-KA; (**b**) fluorescent intensity variation curve traced by IVIS at different time interval. (*n* = 3, * *p* < 0.05 compared with dye solution, TAMRA).

Pictures of the eyes from mice treated with variant eye drops with dye are shown in Figure 5a, quantification data is revealed in Figure 5b showing the percentage of fluorescent signal compared with the initial intensity. After exposure to the eye drops for 5 min, the mice treated with the GNP (67.45% ± 0.56%, * *p* < 0.05 compared with TAMRA) and GNP-KA (72.61% ± 13.43%) exhibited higher fluorescent content retention on eye than mice treated with the TAMRA solution (42.23% ± 17.25%). Subsequently, the highest fluorescence intensity was still recorded in the GNP and GNP-KA groups, and all of the fluorescence intensity in the nano-groups were much higher than the fluorescence intensity in the TAMRA (dye solution) treated mice after 120 min (Figure 5a). At 120 minus, the fluorescence intensity was still around 22% in GNP and GNP-KA group which’s around 3 times higher than the TAMRA treated one (around 6% fluorescence intensity) comparing with the initial dosing intensity.

### 3.5. The Effectiveness of the GNP-KA for Treating Corneal Neovascularization

**Figure 6:**
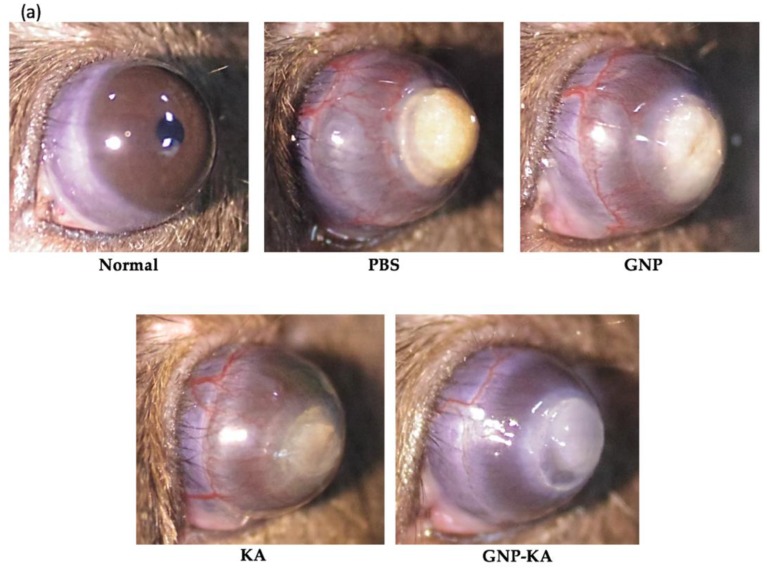

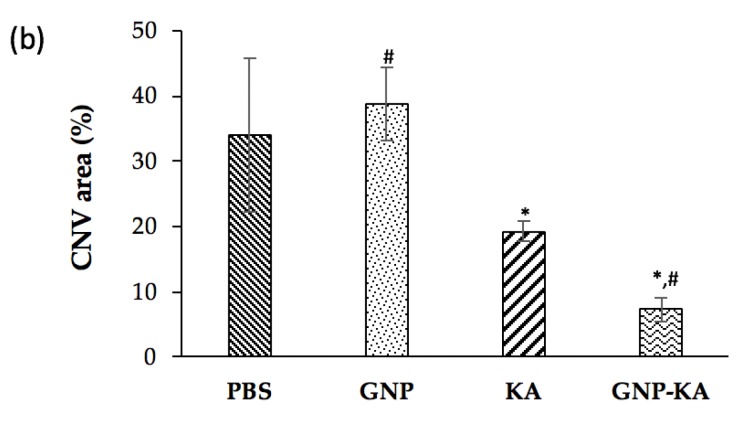
Angiogenesis inhibition in alkali burned cornea. Eyes were treated by variant formulation for one time daily for 7 days. (**a**) Photos were acquired on day 7 in normal cornea, treated by PBS, GNP, KA and GNP-KA group (**b**) Ratio of vessels area in the total cornea was counted. (*n* = 10, mean ± SD). KA concentration: 7.4 μg/mL, gelatin concentration: 150 μg/mL, * *p* < 0.05 compared with PBS, # *p*< 0.05 compared with KA).

The normal mouse eye with smooth and transparent cornea is shown in Figure 6a. Chemical cauterization results in neovascularization from limbus towards the burn scar, which these newly formed capillaries can be obviously seen arising from limbus area and surrounding the burnt white patch in the PBS and GNP treated eyes. In contrast, fewer and thinner visible vessels were observed in both KA and GNP-KA groups (Figure 6a), and the cornea of mice in GNP-KA treated group were better in transparency with the least amount of vessel formation. The amounts of vessel and areas of NV from these images were quantified and represented as CNV ratio (Figure 6b). Treatment of KA contained eye drops (KA solution, or GNP-KA) showed a lower NV ratio indicating a better therapeutic condition for inhibit vessel formation. There is a significant difference between KA (19.1% ± 1.6%) and GNP-KA (8.7% ± 1.9%) treated groups showing the advantages for using nano-formulation as eye drops for effectively treating corneal NV by a single dosage per day.

### 3.6. Histological Results

**Figure 7:**
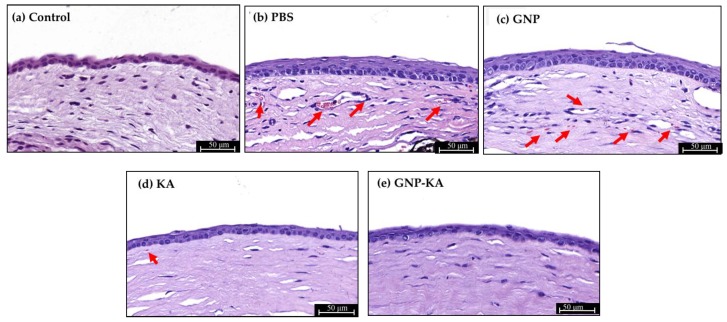
Representative images of the central corneal section with H & E staining. After 7 days treatment, mice corneas were removed for histological section. The vessels and red bloods cells were observed (red arrows) from the corneal section. Groups: (**a**) normal, (**b**) PBS, (**c**) GNP, (**d**) KA, and (**e**) GNP-KA.

The microstructure of the corneas before and after treatment were examined under microscope. Representative images of central corneal sections are shown in Figure 7. From the results, it is evident that there are more vascular tissues with red blood cells observed (red arrows) in the eyes treated with PBS and GNP. Corneal stromal injuries were also observed, confirmed by the looser structure in stroma (Figure 7b,c,d). In the KA group, less blood vessel formation was observed whereas the GNP-KA group exhibited almost entirely avascular tissue with the corneal stroma similar as the control (normal) cornea. These results indicate that kaempferol loaded in gelatin nanoparticles (GNP-KA) have a superior therapeutic effect to inhibit vessels formation in damaged cornea.

### 3.7. Variation of Angiogenic Factors in Cornea

**Figure 8:**
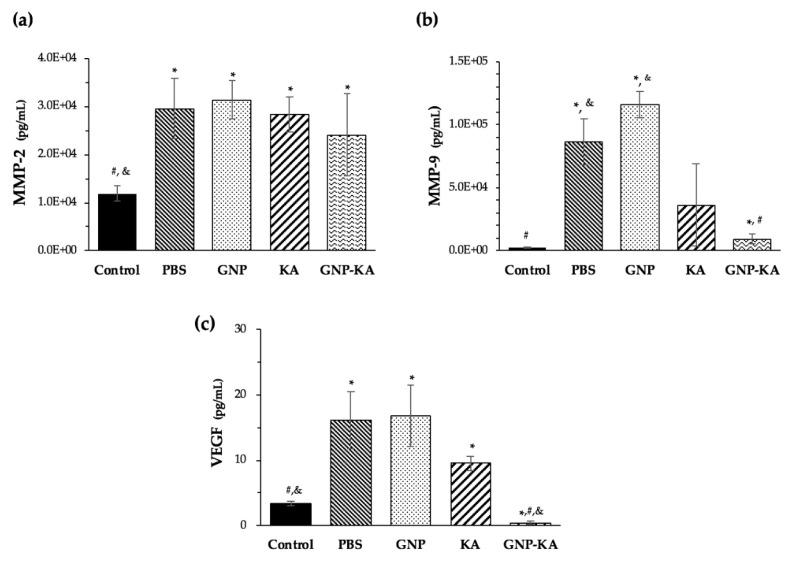
Angiogenetic cytokines variation after 7 days treatment by variant eye drops, the concentration of (**a**) MMP-2, (**b**) MMP-9 and (**c**) VEGF quantification from the cornea lysates. (*n* = 3, mean ± SD, * *p* <0.05 compared with control, # *p* < 0,05 compared with PBS, & *p* < 0,05 compared with KA).

Formation of corneal neovascularization (NV) is closely associated with the accumulation of MMP-2 and MMP-9. Normal corneas were used as control. In Figure 8, the MMP-2/MMP-9 concentration of control group is 11,908 ± 1623 pg/mL and 2616 ± 480 pg/mL, and the VEGF content is 3.42 ± 0.33 pg/mL. Mice corneas with NV treated by PBS showed higher concentration of MMP-2, -9 (29,481 ± 6245/86,479 ± 18,287 pg/mL, * *p* < 0.05 compared with control) and VEGF (16.12 ± 4.34 pg/mL, * *p* < 0.05). GNP has no effect on vessels formation, with variations in angiogenetic concentration having similar tendency as the one treated with PBS (* *p* < 0.05). The KA solution can reduce the concentration of MMP-2 (28,367 ± 3698 pg/mL), MMP-9 (36,243 ± 3231 pg/mL, * *p* < 0.05 compared with control) and VEGF (9.58 ± 1.09 pg/mL, * *p* < 0.05), but no significantly difference with PBS (Figure 8b,c). The nano-KA formula, GNP-KA, can significantly reduce MMP-9 (9386 ± 3946 pg/mL, * *p* < 0.05/# *p* < 0.05 compared with control/PBS) and VEGF (0.45 ± 0.2 pg/mL, * *p* < 0.05/# *p* < 0.05/& *p* < 0.05 compared with control/PBS/KA) content. The concentration of MMP-2 in GNP-KA group (24,101 ± 8511 pg/mL) was the lowest one in all treated groups (excluding the normal cornea). The VEGF content in GNP-KA treated cornea was even lower than the control group (3.42 pg/mL vs. 0.45 pg/mL, Figure 8c). The GNP-KA contained eye drop can effectively reduce the angiogenetic factors (MMP-2/-9, VEGF) and subsequently, reduce vessel formation in the cornea.

Studies had confirmed kaempferol (KA) could inhibit tumor growth, proliferation, and angiogenesis by decreasing VEGF expression in human ovarian cancer cell lines [17,35,36]. New application of KA in eyes was investigated recently. The retinal pigment epithelium (RPE) injury plays an important role in the prevention or delaying age-related macular degeneration (AMD), or diabetic retinopathy etc. The in vitro and in vivo results from Du W’s study revealed that KA could protect RPE cells from oxidative stress damage [37]. As a result, KA has the potential to treat retinal degenerative diseases. The results from Lin C’s study revealed that KA could be used for the treatment of glaucoma due to its anti-inflammatory and anti-oxidative properties. KA can prevent changes in retina thickness and retinal ganglion cell (RGC) death in I/R mice; and pro-inflammatory cytokines were also prevented. Therefore, KA attenuated RGC cell death by inflammasomes, thus leading to a reduction in acute glaucoma [38]. Novel formula of KA such as a nano-form was also developed. The poly (dl-lactic acid-*co*-glycolic acid) (PLGA) nanoparticles (NPs) incorporating KA was studied, and revealed the PLGA-KA NPs had selective toxicity against cancer cells and normal cells, therefore being eligible to be used as a prevention and treatment agent for ovarian cancers [36]. Moreover, Ilk S et al. used lecithin/chitosan (LC) to prepare KA loaded NPs as an antifungal agent. The KA was success-fully encapsulated in LC-NPs with an efficiency of 94% and good physicochemical stability [39]. In this study, the loading rate of KA in gelatin nanoparticles (GNP-KA) was compatible (95%, Table 3) with the KA loaded LC-NPs. The KA-LC NPs exhibited a significantly inhibition efficacy (67%) against *Fusarium oxysporium* after 60 day, exhibiting its slow release capacity. The results indicated that KA-LC-NP formulation could solve problems related to the solubility and loss of KA during use and storage [39]. The storage stability of GNP-KA can last for four weeks at 4 °C (data not shown), the slow release capacity of KA from GNPs can also be confirmed by the cell viability and migration test showing the longer effect for caused cell viability reduced after 3 days (Figure 3) and migration inhibition lasting for 24 h (Figure 4). From the details above, new application of KA and variant polymeric based nanoparticles for loading KA were synthesized for application in cancer, anti-angiogenesis, and antifungal treatment [17,36,37,39,40]. But none of the KA related nanomedicine was studied in eye treatment. In this study, we proved that kaempferol loaded in gelatin nanoparticles (GNP-KA) can effectively inhibit HUVEC cells function in vitro (Figure 3 and Figure 4) and retard vessel formation in mice cornea (Figure 6, Figure 7 and Figure 8) via a decrease in the concentration of MMP-2,-9 and VEGF. 

## 4. Conclusions

In conclusion, it was observed through DLS that the size of the nanoparticles carrying the KA averaged from about 80–150 nm, the zeta potential was about 22–26 mV and the PDI ranged from 0.199 to 0.306. TEM confirmed the morphology of such particles to be round and distinct particulates with no aggregation. The successful preparation of KA-loaded GNPs was confirmed by FT-IR through the individual peaks of naphthol phenolic -OH bonded structure from KA. The results obtained from the third day of the WST-8 cell viability tests for GNP-KA established the significant cytostatic activity of the successfully prepared NPs by displaying the inhibition of HUVECs cell viability compared to the KA group. On the other hand, the Live/Dead test, also reiterated the inhibitory activity of the cells. Cell migration tests exhibited the effective inhibition of cell migration of the HUVECs in GNP-KA compared to the other groups. The GNP-KA NPs also showed good therapeutic effect based on the fact that the GNP-KA group could reduce blood vessel growth on the cornea. Also, it was implied through H&E staining that the GNP-KA group led to a common structure and less vascular tissue observation on the cornea compared to the other test groups. These results indicated that the GNP-KA NPs formulation could have significant therapeutic effects against corneal neovascularization and also exhibit anti-angiogenic effects, thus making it a viable candidate for utilizing as an eye drops for effective treating cornea neovascularization. 

## Figures and Tables

**Table 1:** Characterization of GNP-KA prepared in variant glutaraldehyde (GA) concentration.

GA Concn.	Size (nm)	Zeta (mV)	PDI	EE (%)	LR (%)
0.1 (*v*/*v* %)	281 ± 16	+21.5 ± 0.5	0.199 ± 0.020	85 ± 10	2.1 ± 0.3
0.4 (*v*/*v* %)	148 ± 10	+24.4 ± 1.9	0.203 ± 0.032	96 ± 2	2.4 ± 0.1

2% (*w*/*v*) gelatin solution, stirred for 3 h, *n* = 3.

**Table 2:** Characterization of GNP-KA prepared in variant GA cross-linking time.

Crosslinking Time	Size (nm)	Zeta (mV)	PDI	EE (%)	LR (%)
1 h	138 ± 3	+22.8 ± 0.7	0.330 ± 0.032	90 ± 2	2.2 ± 0.1
3 h	149 ± 10	+24.4 ± 1.9	0.203 ± 0.031	96 ± 2	2.4 ± 0.1
16 h	90 ± 8	+21.4 ± 0.1	0.275 ± 0.010	98 ± 1	2.4 ± 0.0

2% (*w*/*v*) gelatin solution, 0.4% GA, *n* = 3.

**Table 3:** Characterization of GNP-KA prepared in variant gelatin concentration.

Gelatin Concn.	Size (nm)	Zeta (mV)	PDI	EE (%)	LR (%)
1% (*w*/*v*) *	133 ± 15	+26.6 ± 1.4	0.211 ± 0.072	/	/
1% (*w*/*v*)	85 ± 8	+25.6 ± 2.1	0.306 ± 0.051	95 ± 1	4.6 ± 0.1
2% (*w*/*v*)	149 ± 10	+24.4 ± 1.9	0.203 ± 0.030	96 ± 2	2.4 ± 0.1

* GNP (without KA addition), 0.4% GA, stirred for 3 h. *n* = 3.
